# Functional Studies of Five Toxin-Antitoxin Modules in *Mycobacterium tuberculosis* H37Rv

**DOI:** 10.3389/fmicb.2016.02071

**Published:** 2016-12-21

**Authors:** Yoonji Kim, Eunsil Choi, Jihwan Hwang

**Affiliations:** Department of Microbiology, Pusan National UniversityBusan, Republic of Korea

**Keywords:** toxin, antitoxin, VapBC, MazEF, *Mycobacterium tuberculosis*

## Abstract

Toxin–antitoxin (TA) systems, which consist of an intracellular toxin and its antidote (antitoxin), are encoded by ubiquitous genetic modules in prokaryotes. Commonly, the activity of a toxin is inhibited by its antitoxin under normal growth conditions. However, antitoxins are degraded in response to environmental stress, and toxins liberated from antitoxins consequently induce cell death or growth arrest. In free-living prokaryotes, TA systems are often present in large numbers and are considered to be associated with the adaptation of pathogenic bacteria or extremophiles to various unfavorable environments by shifting cells to a slow growth rate. Genomic analysis of the human pathogen *Mycobacterium tuberculosis* H37Rv (*Mtb*) revealed the presence of a large number of TA systems. Accordingly, we investigated five uncharacterized TA systems (Rv2019-Rv2018, Rv3697c-Rv3697A, Rv3180c-Rv3181c, Rv0299-Rv0298, and Rv3749c-Rv3750c) of *Mtb*. Among these, the expression of the Rv2019 toxin inhibited the growth of *Escherichia coli*, and *M. smegmatis* and this growth defect was recovered by the expression of the Rv2018 antitoxin. Interestingly, Rv3180c was toxic only in *M. smegmatis*, whose toxicity was neutralized by Rv3181c antitoxin. *In vivo* and *in vitro* assays revealed the ribosomal RNA (rRNA) cleavage activity of the Rv2019 toxin. Moreover, mRNAs appeared to be substrates of Rv2019. Therefore, we concluded that the ribonuclease activity of the Rv2019 toxin triggers the growth defect in *E. coli* and that the Rv2018 antitoxin inhibits the ribonuclease activity of the Rv2019 toxin.

## Introduction

Toxin–antitoxin (TA) systems are encoded by ubiquitous genetic modules as an operon in prokaryotes and consist of an intracellular toxin and its antidote (antitoxin) (Ramage et al., 2009). Previously, these systems were known as plasmid addiction modules of large plasmids with low copy numbers (Van Melderen et al., 1994). The control of cell death (*ccd*) system was first identified as a TA system in the *Escherichia coli* F plasmid (Jaffe et al., 1985); it is organized as an operon that contains two genes encoding a toxic protein (CcdB toxin) and an antidote protein (CcdA antitoxin). When cells are carrying the F plasmid after division, the *ccdB* toxin and *ccdA* antitoxin are simultaneously expressed and form a stable TA (CcdB–CcdA) complex that neutralizes toxin activity. However, when the F plasmid is lost by chance during cell division, the CcdA antitoxins remaining in plasmid-free daughter cells are rapidly degraded by the Lon protease. Consequently, CcdB toxins released from the CcdB–CcdA complex interact with their target, DNA gyrase, leading to double-strand DNA breaks and ultimately to cell death. Similarly, other plasmid-borne TA systems have been identified as post-segregational killing systems (Gerdes et al., 1990; Jiang et al., 2002) that play a particularly important role in the maintenance of virulence plasmids in pathogenic bacteria (Hayes, 2003; De la Cruz et al., 2013). However, numerous TA systems have recently been discovered; these are encoded by prokaryotic genomes and are commonly considered stress-responsive genetic modules (Sevin and Barloy-Hubler, 2007; Shao et al., 2011).

At present, TA systems are classified into five types according to the nature and mode of action of the antitoxin that counteracts the activity of the toxin (Unterholzner et al., 2013). Toxins are proteins, and antitoxins are either proteins (Types II, IV, and V) or small RNAs (Types I and III) (Schuster and Bertram, 2013). In the Type I system, RNA antitoxins that are complementary to toxin transcripts bind to toxin mRNAs and arrest the translation of toxin mRNAs (Gerdes et al., 1990; Vogel et al., 2004; Weaver et al., 2004; Kawano et al., 2007; Fozo et al., 2008). The toxins and antitoxins of Type II systems are proteins, and most toxins function as endoribonucleases that target rRNA, mRNA, or tRNA. The antitoxin directly associates with its cognate toxin to antagonize the activity of the latter (Jiang et al., 2002; Zhang Y. et al., 2003; Munoz-Gomez et al., 2005; Liu et al., 2008; Jorgensen et al., 2009; Schumacher et al., 2009; Hallez et al., 2010; Winther and Gerdes, 2011; Zhang and Inouye, 2011; Brzozowska and Zielenkiewicz, 2013). In the Type III system, the toxin protein is inactivated by binding to RNA antitoxin fragments cleaved by the ribonuclease of the toxin (Fineran et al., 2009; Blower et al., 2012). Unlike the Type II system, toxins and antitoxins of the Type IV system do not interact directly, and antitoxins protect the target of the toxin, reversing the effect of the toxin (Masuda et al., 2012a,b). In the Type V system, the ribonuclease activity of the antitoxin can degrade the toxin mRNA (Wang et al., 2012).

Surprisingly, a relatively enormous number (79) of TA systems have been identified in the human pathogen *Mycobacterium tuberculosis* H37Rv (*Mtb*), and notably, unlike the related non-pathogenic mycobacterial species, members of the *M. tuberculosis* complex, *M. microti, M. bovis, M. africanum*, and *M. canetti*, encode an equally large number of TA systems (66, 65, 65, and 60, respectively) (Ramage et al., 2009). Considering this conservation, the TA systems of pathogenic Mycobacteria may be related to their pathogenesis, persistence, virulence, and biofilm formation. Therefore, to investigate the important role of the TA system of *Mtb*, we selected 4 uncharacterized TA systems of the VapBC TA family (Rv2019-Rv2018, Rv3697c-Rv3697A, Rv3180c-Rv3181c, and Rv3749c-Rv3750c) as well as 1 TA system homologous to the MazEF TA family (Rv0299-Rv0298). Among these, we showed that expression of the Rv2019 toxin inhibited the growth of *E. coli* and that this growth defect was recovered by expression of the Rv2018 antitoxin. *In vitro* studies showed that the ribosomal RNA (rRNA) cleavage activity of the Rv2019 toxin requires a divalent metal ion. Therefore, we concluded that the ribonuclease activity of the Rv2019 toxin triggers a growth defect in *E. coli* and that the Rv2018 antitoxin inhibits the ribonuclease activity of the Rv2019 toxin.

## Materials and Methods

### Bacterial Strains and Growth Conditions

The bacterial strains used in this study are listed in **Table 1**. For all studies, bacterial strains were grown with aeration at 37°C. *E. coli* BL21(DE3) was grown in LB or M9 minimal media supplemented with casamino acids and 0.2% glucose or 0.2% glycerol. *M. smegmatis* mc^2^ 155 was grown in Middlebrook 7H9 (a liquid medium, Difco) containing 0.2% glycerol and 0.02% Tween 80 or Middlebrook 7H10 (a solid agar medium, Difco) containing 0.2% glycerol. Chloramphenicol (Cm) or ampicillin (Amp) was added to a concentration of 50 μg/ml to cultures of *E. coli*, and kanamycin was added to a concentration of 50 μg/ml to cultures of *E. coli* and 25 μg/ml to cultures of *M. smegmatis*.

**Table 1:** Bacterial strains and plasmids used in this study.

Strains/Plasmids	Genotypes	Reference
BW25113	*E. coli*, F-,Δ(*araD-araB*)*567*,Δ*lacZ4787*(::rrnB-3), λ-, *rph-1*,Δ(*rhaD-rhaB*)*568, hsdR514*	*E. coli* Genetic Resources at Yale CGSC
BL21(DE3)	*E. coli*, F-, *lon-11*,Δ(*ompT-nfrA*)*885*,Δ(*galM-ybhJ*)*884*,λ*DE3* [*lacI, lacUV5-T7 gene 1, ind1, sam7, nin5*], Δ*46*, [*mal*^+^]*_K-12_*(λ^S^), *hsdS10*	*E. coli* Genetic Resources at Yale CGSC
*M. smegmatis* mc^2^155	*M. smegmatis, ept-1*	Snapper et al., 1988
pBAD33	*araBAD* promoter, pACYC184 *ori*, Cm^R^	Guzman et al., 1995
pET21c	T7 promoter, pBR322 *ori*, Amp^R^	Novagen
pGST	T7-lac promoter, pBR322 *ori*, Kan^R^	Ogawa et al., 1996
pMV306AC	Acetamide promoter, *oriE*, Kan^R^, *E. coli*-*Mycobacterium* shuttle vector	Stover et al., 1991
pBAD33-Rv2019	*Rv2019*^+^ (toxin), pBAD33	This study
pBAD33-Rv2019_D4K_	*Rv2019_D4K_* (toxin), pBAD33	This study
pBAD33-Rv2019_E41K_	*Rv2019_E41K_* (toxin), pBAD33	This study
pBAD33-Rv2019_D98K_	*Rv2019_D98K_* (toxin), pBAD33	This study
pBAD33-Rv3697c	*Rv3697c*^+^ (toxin), pBAD33	This study
pBAD33-Rv3180c	*Rv3180c*^+^ (toxin), pBAD33	This study
pBAD33-Rv0299	*Rv0299*^+^ (toxin), pBAD33	This study
pBAD33-Rv3749c	*Rv3749c*^+^ (toxin), pBAD33	This study
pBAD33-Rv2018	*Rv2018*^+^ (antitoxin), pBAD33	This study
pET21c-Rv2019	*Rv2019*^+^ (toxin), pET21c	This study
pET21c-Rv2019His	*Rv2019*^+^ (toxin), pET21c, C-terminal His-tag	This study
pET21c-Rv2018	*Rv2018*^+^ (antitoxin), pET21c	This study
pET21c-Rv3697A	*Rv3697A*^+^ (antitoxin), pET21c	This study
pET21c-Rv3181c	*Rv3181c*^+^ (antitoxin), pET21c	This study
pET21c-Rv0298	*Rv0298*^+^ (antitoxin), pET21c	This study
pET21c-Rv3750c	*Rv3750c*^+^ (antitoxin), pET21c	This study
pGST-Rv2019	*Rv2019*^+^ (toxin), pGST	This study
pGST-Rv3697c	*Rv3697c*^+^ (toxin), pGST	This study
pGST-Rv3180c	*Rv3180c*^+^ (toxin), pGST	This study
pGST-Rv0299	*Rv0299*^+^ (toxin), pGST	This study
pGST-Rv3749c	*Rv3749c*^+^ (toxin), pGST	This study
pMV306AC-Rv2019	*Rv2019*^+^ (toxin), pMV306AC	This study
pMV306AC-Rv2018-2019	*Rv2018*^+^ (antitoxin), *Rv2019*^+^ (toxin), pMV306AC	This study
pMV306AC-Rv3697c	*Rv3697c*^+^ (toxin), pMV306AC	This study
pMV306AC-Rv3180c	*Rv3180c*^+^ (toxin), pMV306AC	This study
pMV306AC-Rv3180c-3181c	*Rv3180c*^+^ (toxin), *Rv3181c*^+^ (antitoxin), pMV306AC	This study
pMV306AC-Rv0299	*Rv0299*^+^ (toxin), pMV306AC	This study
pMV306AC-Rv3749c	*Rv3749c*^+^ (toxin), pMV306AC	This study

*Amp^R^, ampicillin resistance; Kan^R^, Kanamycin resistance; Cm^R^, Chloramphenicol resistance.*

### Plasmid Construction

To construct plasmids, toxin and antitoxin genes were amplified from *Mtb* genomic DNA by polymerase chain reaction (PCR). The amplified PCR fragments were subcloned into the *Sma*I site of pUC19, and the sequences of the amplicons were verified using DNA sequencing. Using DNAs digested by the appropriate restriction enzymes, toxin genes were subsequently cloned into the *Nde*I-*Hind*III sites (for *Rv2019, Rv3180c, Rv3697c*, or *Rv0299*) or *Nde*I-*Xba*I sites (for *Rv3749c*) of the pBAD33 vector containing pA15 origin of replication (∼20 copies per cell) the arabinose-inducible (*araBAD*) promoter (Guzman et al., 1995) to generate pBAD33-Rv2019, pBAD33-Rv3180c, pBAD33-Rv3697c, pBAD33-Rv0299, and pBAD33-Rv3749c. Antitoxin genes were cloned into the *Nde*I-*Eco*RI sites of the pET21c vector containing an isopropyl β-D-1-thiogalactopyranoside (IPTG)-inducible T7 promoter to generate pET21c-Rv2018, pET21c-Rv3181c, pET21c-Rv3697A, pET21c-Rv0298, and pET21c-Rv3750c.

To construct a plasmid expressing a chimeric glutathione *S*-transferase (GST) Rv2019 protein (pGST-Rv2019), pET21c-Rv2019 was digested with *Nde*I and *Xho*I, and the *Rv2019* fragment was ligated into the *Nde*I-*Xho*I sites of the pGST vector containing an IPTG-inducible T7-*lac* promoter. To express the toxins in *M. smegmatis*, pUC19-toxin subclones were digested with *Nde*I and *Xba*I, and the resulting fragment was ligated into the *Nde*I-*Xba*I sites of the pMV306AC vector containing an acetamide-inducible promoter (Stover et al., 1991). To construct Rv2019 mutant clones, the primers listed in Supplementary Table S1 were used to amplify gene fragments, and the resulting DNA fragments harboring mutation were inserted into pBAD33, yielding pBAD33-Rv2019_D4K_, pBAD33-Rv2019_E41K_, and pBAD33-Rv2019_D98K_. All plasmids used in this study are listed in **Table 1**.

### Rv2019-Rv2018 Neutralization Assay

Overnight cultures of BL21(DE3) harboring compatible plasmid pairs (pBAD33 and pET21c, pBAD33-Rv2019 and pET21c, pBAD33 and pET21c-Rv2018, or pBAD33-Rv2019 and pET21c-Rv2018) were diluted 10^2^-fold in fresh M9 minimal media containing Cm and Amp, and the diluted cultures were grown to the exponential phase at 37°C. The cells were harvested by centrifugation and resuspended in M9 minimal media to an optical density at 600 nm (OD_600_) = 0.2. The resuspension was diluted 10^-1^, 10^-2^, 10^-3^, and 10^-4^-folds, and each diluted sample was spotted onto M9 agar plates containing Cm and Amp with 0.2% L-arabinose, 0.05 mM IPTG, or both or without inducers. The plates were incubated overnight at 37°C. For a neutralization assay in liquid culture, BL21(DE3) harboring pBAD33-Rv2019 and pET21c-Rv2018 were grown at 37°C to the exponential phase in M9 minimal media containing Cm and Amp. The cultures were diluted fivefold in the same minimal media, and inducers were added as follows: 0.2% L-arabinose, 0.05 mM IPTG, or both. The diluted cultures were incubated at 37°C for 2 h and continuously diluted fivefold in fresh M9 minimal media containing inducers every 2 h, and OD_600_ was measured.

### Purification of GST and GST-Rv2019

Overnight cultures of BL21(DE3) transformed with pGST or pGST-Rv2019 were diluted 50-fold in 1 L of fresh LB media containing kanamycin and incubated at 37°C until OD_600_ reached 0.5–0.6. IPTG was added to 0.5 mM, and the cultures were further incubated with shaking (200 rpm) at 37°C for 2 h. The cells were centrifuged for 20 min at 4,000 × *g*, and the cell pellet was washed in 30 ml of 20 mM Tris–HCl (pH 6.8). The washed cells were centrifuged again and resuspended in 20 ml (for GST) or 66 ml (for GST-Rv2019) of lysis buffer [50 mM Tris–HCl (pH 8.0), 100 mM NaCl, 5 mM β-mercaptoethanol] with (for GST-Rv2019) or without (for GST) sarkosyl (*N*-lauroylsarcosine). The resuspended cells were lysed using a sonicator. Lysates were centrifuged for 25 min at 10,000 × *g*, and the supernatant was centrifuged for 1 h at 180,000 × *g* in a Beckman 70Ti rotor. The final supernatant was loaded onto a glutathione agarose column that was equilibrated with 10 column volumes of lysis buffer. The column was washed with 30 column volumes of lysis buffer, and bound proteins were eluted with 10 1-ml aliquots of elution buffer containing 10 mM reduced glutathione. The eluted proteins were dialyzed twice overnight against 2 L of storage buffer [50 mM Tris–HCl (pH 8.0), 100 mM NaCl].

### Extraction of Total RNA

The hot phenol method was used to extract total cellular RNA (Sarmientos et al., 1983). BL21(DE3) was grown to OD_600_ = 1.0, and 1.5 ml of the culture was centrifuged for 2 min at 17,000 × *g*. Pelleted cells were resuspended in 500 μl of solution A (20 mM sodium acetate, 10 mM EDTA, 0.5% SDS), and an equal volume of hot phenol was added, followed by incubation for 10 min at 60°C. After centrifugation for 10 min at 17,000 × *g*, 400 μl of the aqueous phase was mixed with 40 μl of 3 M sodium acetate (pH 4.3). In total, 1 ml of ice-cold ethanol was added to the mixture, which was incubated for 30 min at -20°C. RNA was collected by centrifugation for 10 min at 17,000 × *g*. The pellet was resuspended in 200 μl of solution B (20 mM sodium acetate, 10 mM EDTA) and mixed with 20 μl of 3 M sodium acetate (pH 4.3). Next, 1 ml of ice-cold ethanol was added, and the sample was incubated for 30 min at -20°C. RNA was collected by centrifugation for 10 min at 17,000 × *g*. The pellet was washed with 200 μl of 75% ethanol and dissolved in 50 μl of distilled water. The quality of RNA was assessed using agarose gel electrophoresis, and the RNA concentration was determined using Thermo Scientific NanoDrop 2000.

### rRNA Cleavage Assay

For the *in vivo* assay, BL21(DE3) harboring pBAD33 or pBAD33-Rv2019 was grown to OD_600_ = 0.6–0.7 in M9 minimal media containing Cm. The cultures were diluted fivefold in fresh M9 media supplemented with 0.2% L-arabinose, and the diluted cultures were incubated at 37°C for 2 h. Cultures were continuously diluted fivefold in fresh M9 minimal media containing inducer every 2 h. After induction for 0, 2, 4, or 6 h, equal numbers of cells were harvested according to OD_600_, and the pellets were resuspended in 100 μl of 20 mM Tris–HCl (pH 6.8). An equal volume of phenol (pH 8.0) was added, which was vortexed to lyse the cells, and the aqueous phase was electrophoresed through 1.2% agarose gel. For the *in vitro* assay, total RNAs were extracted from BL21(DE3), as described earlier. The reaction buffer containing 50 mM Tris–HCl (pH 8.0) and the purified protein (15 pmole of GST or GST-Rv2019) was incubated with 2 μg of RNA at 37°C. At the indicated times, reactions were stopped by adding 3 μl of 6x RNA loading buffer [10 mM Tris–HCl (pH 7.5), 0.03% bromophenol blue, 0.03% xylene cyanol FF, 60% glycerol, and 60 mM EDTA]. The samples were electrophoresed through 1.2% agarose gel, and RNAs were visualized using ethidium bromide.

### Quantitative Real-Time PCR (qRT-PCR)

BL21(DE3) harboring pBAD33 or pBAD33-Rv2019 was grown to the exponential phase, and the culture was diluted 10-fold in fresh M9 minimal medium containing Cm. L-arabinose was added to the diluted culture to a final concentration of 0.2%. Next, the cultures were incubated with shaking (200 rpm) at 37°C for 4 h; the cultures were then diluted 10-fold in fresh M9 minimal media containing Cm and 0.2% L-arabinose and incubated at 37°C. After 8 h, the same amounts of cells were harvested, and total RNAs were extracted using the hot phenol method, as described above. RNase-free DNase (Qiagen) was treated to remove contaminating genomic DNA, according to the manufacturer’s instructions. After DNase treatment, the quality of total RNA was assessed using agarose gel electrophoresis, and the RNA concentration was determined using Thermo Scientific NanoDrop 2000. Complementary DNA (cDNA) was synthesized using the Superscript III First Strand Synthesis for RT-PCR Kit (Invitrogen) with 1 μg of total RNA. The StepOne Plus Real-time PCR System (Applied Biosystems) was used to amplify 1 μl of cDNA added to SYBR Green master mix (Qiagen). The eight pairs of primers used for qRT-PCR are listed in Supplementary Table S1. Thermocycling conditions were as follows: initial denaturation for 15 min at 95°C, followed by 40 cycles at 95°C for 5 s, 55°C or 59°C (only for reaction using the glyS2 primers) for 30 s, and 72°C for 50 s.

### Construction of *M. smegmatis* Strains Carrying Genomic Expression Cassettes Encoding Toxins

The shuttle vector pMV306AC lacking a mycobacterial origin of replication was used for integration, which occurs between the *attP* site of the vector and the *attB* site of *M. smegmatis* genomic DNA (Stover et al., 1991). *Rv2019, Rv2018-2019, Rv3180c, Rv3180c-3181c, Rv3697c, Rv3749c*, or *Rv0299* was each cloned into the *Nde*I-*Xba*I sites of the acetamide-inducible pMV306AC vector to generate pMV306AC-Rv2019, pMV306AC-Rv2018-2019, pMV306AC-Rv3180c, pMV306AC-Rv3180c-3181c, pMV306AC-Rv3697c, pMV306AC-Rv3749c, or pMV306AC-Rv0299, respectively. Electroporation was used to introduce the plasmids into *M. smegmatis* mc^2^ 155 (Snapper et al., 1990). Single colonies of transformed cells were selected by plating the cultures onto agar plates containing kanamycin. Colony PCR was performed to confirm the integration of the plasmid into the bacterial genome (data not shown).

## Results

### Assessment of the Toxic Effects of 5 *Mtb* TA Systems on the Growth of *E. coli*

The *Mtb* genome encodes 79 TA systems as follows: 10 MazF homologs, 50 VapC homologs, 2 RelE homologs, 3 HigB homologs, 2 ParE homologs, 1 YoeB homolog, and 11 putative TA systems (Sala et al., 2014). Notably, because of the numerous TA systems in *Mtb*, some TA modules were characterized to identify their TA functions; however, the functional mechanisms of most *Mtb* TA systems are unknown. Therefore, in the present study, in order to elucidate functions of those TA systems, we selected 4 VapBC TA systems (Rv2019-Rv2018, Rv3697c-Rv3697A, Rv3180c-Rv3181c, and Rv3749c-Rv3750c) and 1 MazEF TA system (Rv0299-Rv0298) of *Mtb* whose toxicities are still unknown in *Mtb* as well as in *E. coli*.

**FIGURE 1:**
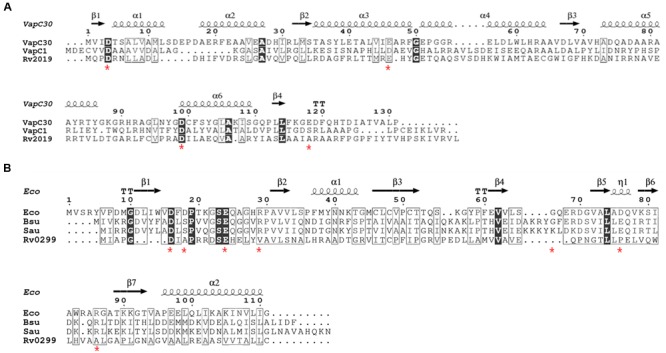
**Amino acid sequence alignments of VapC or MazF homologs. (A)** Sequence alignments of Rv2019 with VapC homologs VapC30, *Mtb* VapC30 (GenBank accession number NP_215138.1); VapC1, *Mtb* VapC1 (CCP42788.1); and Rv2019, *Mtb* Rv2019 (NP_216535.1). The numbers correspond to amino acid residue numbers and asterisks indicate four highly conserved acidic residues in VapC30. **(B)** Sequence alignments of Rv0299 with MazF homologs. Eco, *Escherichia coli* MazF (GenBank accession number CDZ21577.1); Bsu, *Bacillus subtilis* MazF (P96622.1); Sau, *Staphylococcus aureus* MazF (AKB00150.1); and Rv0299, *Mtb* Rv0299 MazF (CCP43029.1). Asterisks indicate seven amino acids residues in *E. coli* MazF that bind substrate. Identical and similar residues are enclosed in black and white boxes, respectively.

VapC toxin homologs share sequence similarities with the PIN domain, which is a *Pi*lT *N*-terminal domain (Arcus et al., 2011). The three-dimensional structure of the active site of PIN domain includes four highly conserved acidic residues that coordinate the divalent metal ion. Notably, a cluster of metal ion-associated acidic residues forms a cavity and plays an important role in ribonuclease activity (Das et al., 2014). First, we compared the amino acids sequences of four VapC homologs with those of *Mtb* VapC1 and VapC30, each of which contains well-conserved acidic residues. As shown in **Figure 1A**, the alignment of the amino acids sequence of the Rv2019 toxin with those of the *Mtb* VapC homologs (VapC1 and VapC30) showed relatively low identities (6.87–10.52%) and similarities (10.68–20.61%). Although the overall sequence conservation was poor, the three acidic residues Asp4, Glu41, and Asp98 in Rv2019 were well conserved. However, the fourth acidic residue was not conserved. When the other three VapC homologs (Rv3180c, Rv3697c, and Rv3749c) were aligned with *Mtb* VapC1 and VapC30, three acidic residues were less well conserved (Supplementary Figure S1). All *Mtb* MazF toxins appeared distantly related to known MazF proteins of other bacteria (Ramage et al., 2009), and in the case of Rv0299 toxin as a MazF homolog, the similarity and identity scores of Rv0299 and MazF homologs in *E. coli, Bacillus subtilis*, and *Staphylococcus aureus* were 9.9–12.61% and 5–8%, respectively (**Figure 1B**).

**FIGURE 2:**
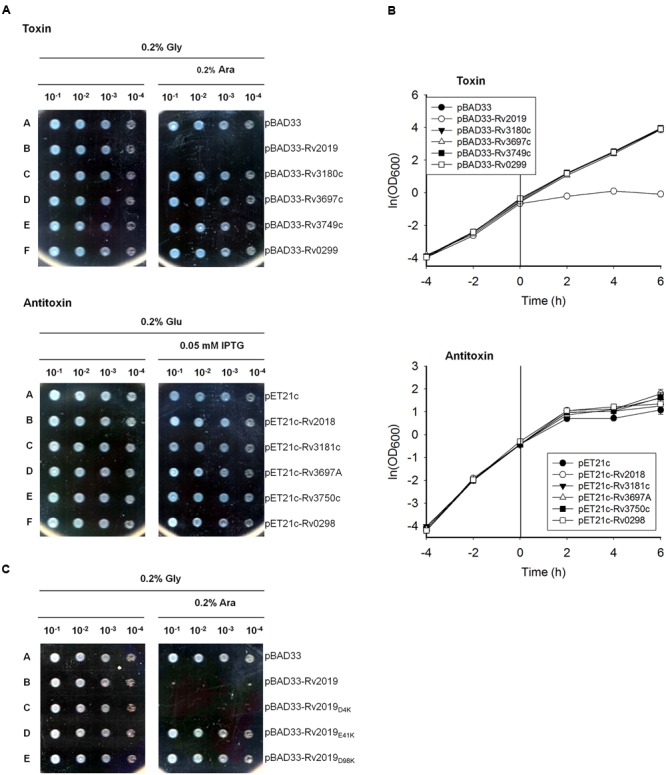
**Phenotypes of five putative toxin-antitoxin systems and mutant Rv2019 proteins. (A)** Inhibition of colony formation of *E. coli* BL21(DE3) expressing Rv2019 toxin. BL21(DE3) harboring plasmid pBAD33, pBAD33-toxin, pET21c, or pET21c-antitoxin was collected during the exponential phase and harvested by centrifugation. The cell pellet was resuspended to an optical density at 600 nm (OD_600_) = 0.2 in M9 minimal media and diluted 10^-1^, 10^-2^, 10^-3^, and 10^-4^ folds. Each diluted sample was spotted on M9 agar plates. **(B)** Growth curves of *E. coli* BL21(DE3) expressing toxin or antitoxin. BL21(DE3) harboring the plasmids pBAD33, pBAD33-toxin, pET21c, or pET21c-antitoxin was diluted during the exponential phase, and the inducer was added at a time defined as 0 h. Cultures were diluted at every 2 h, and OD_600_ was measured. **(C)** Colony formation of *E. coli* BL21(DE3) expressing wild-type and mutant Rv2019 proteins. BL21(DE3) harboring pBAD33, pBAD33-Rv2019, pBAD33-Rv2019_D4K_, pBAD33-Rv2019_E41K_, or pBAD33-Rv2019_D98K_ was spotted on M9 agar plates in a manner similar to that described in **(A)**.

To assess the toxic effects of five putative *Mtb* TA systems on *E. coli*, we transformed BL21(DE3) with the pBAD33-toxin plasmid and induced the expression of toxin genes using 0.2% L-arabinose. The expression of the five mycobacterial TA systems in cells grown on solid M9 media showed only that the expression of Rv2019 inhibited colony formation of BL21(DE3) (**Figure 2A**). Further, the expression of Rv3180c, Rv3697c, Rv3749c, and Rv0299 did not influence colony formation of BL21(DE3). The open reading frames of *Rv2018, Rv3181c, Rv3697A, Rv3750c*, and *Rv0298* were ligated to the pET21c vector containing an IPTG-inducible (T7) promoter. In the presence of 0.05 mM IPTG, the expression of the antitoxins did not affect cell growth, suggesting that the antitoxin proteins were not toxic. Further, only the Rv2019 toxin severely inhibited the growth of *E. coli* when each of the five toxins was expressed in cells cultured in liquid M9 media (**Figure 2B**). These results suggest that *Mtb* Rv2019 functions as a toxin, at least in *E. coli*.

Next, to investigate whether the three acidic residues are indispensable for the toxicity of the Rv2019 toxin, the mutations D4K, E41K, and D98K were individually introduced into the primary sequence of the Rv2019 toxin. In **Figure 2C**, the expression of the Rv2019_D4K_ mutant inhibited colony formation of BL21(DE3) to an extent similar to that of wild-type Rv2019. In contrast, the Rv2019_E41K_ and Rv2019_D98K_ mutants did not inhibit colony formation of BL21(DE3). These results suggest that Glu41 and Asp98, but not Asp4, play a pivotal role in the toxicity of Rv2019 and coordination of metal ion and that the toxicity is solely due to the expression of Rv2019.

### Neutralization of the Rv2019 Toxin by the Rv2018 Antitoxin

**FIGURE 3:**
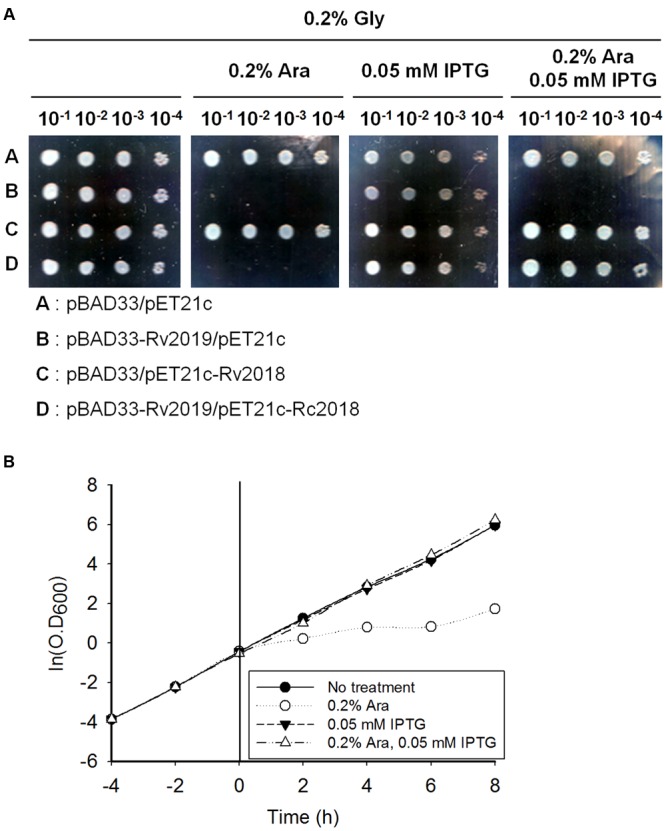
**Neutralization of the toxic effect of Rv2019 by the Rv2018 antitoxin. (A)** Recovered colony formation of BL21(DE3) coexpressing Rv2019 and Rv2018. BL21(DE3) harboring compatible plasmid pairs (pBAD33 and pET21c, pBAD33-Rv2019 and pET21c, pBAD33 and pET21c-Rv2018, or pBAD33-Rv2019 and pET21c-Rv2018) was diluted during the exponential phase. The cells were harvested by centrifugation, resuspended to OD_600_ = 0.2 in M9 minimal media, and diluted 10^-1^, 10^-2^, 10^-3^, and 10^-4^ folds. Each of the diluted samples was spotted on M9 agar plates. **(B)** Growth curve of BL21(DE3) coexpressing Rv2019 and Rv2018. BL21(DE3) harboring pBAD33-Rv2019 and pET21c-Rv2018 was diluted fivefold in fresh M9 minimal media during the exponential phase, and inducer was added at 0 h. Cultures were continuously diluted fivefold in fresh M9 minimal media containing inducers every 2 h, and OD_600_ was measured.

In exponentially growing healthy cells, all toxins are constitutively expressed and interact with their cognate antitoxins, either protein or RNA molecules, except for the Type IV TA system. This association prevents the function of a toxin by neutralizing its toxicity. Therefore, to examine whether the toxic effect of Rv2019 was neutralized by its cognate Rv2018 antitoxin, *E. coli* BL21(DE3) was cotransformed with compatible plasmid pairs (pBAD33 and pET21c, pBAD33-Rv2019 and pET21c, pBAD33 and pET21c-Rv2018, or pBAD33-Rv2019 and pET21c-Rv2018). In BL21(DE3) cotransformants, the expression of the gene encoding the Rv2019 toxin or the Rv2018 antitoxin was independently induced by 0.2% L-arabinose or 0.05 mM IPTG and coinduced when 0.2% L-arabinose and 0.05 mM IPTG were added together. We found that the expression of the Rv2019 toxin inhibited colony formation of BL21(DE3) in the presence of arabinose (**Figure 3A**), which is consistent with the previous result (**Figure 2A**). However, coexpression of the Rv2019 toxin and the cognate Rv2018 antitoxin recovered normal colony formation of BL21(DE3) in the presence of both inducers. Similar results were obtained using liquid cultures (**Figure 3B**). Furthermore, a reciprocal exchange in expression vectors yielded the same results (Supplementary Figure S2A). Thus, these findings demonstrate that the expression of the Rv2019 toxin is toxic to *E. coli* and its cognate antitoxin Rv2018 counteracts the toxic effect of the Rv2019 toxin.

### Ribonuclease Activity of the Rv2019 Toxin *In vivo*

**FIGURE 4:**
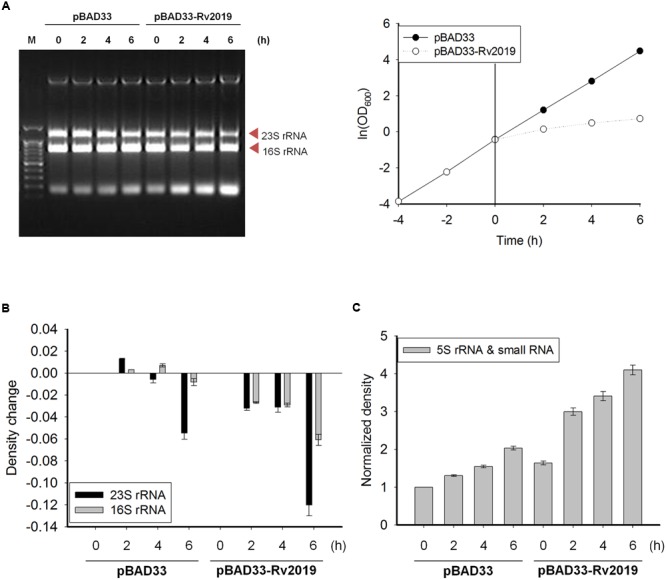
**Ribosomal RNA cleavage activity of Rv2019 *in vivo*. (A)** Ribonuclease activity of Rv2019. BL21(DE3) harboring pBAD33 or pBAD33-Rv2019 was cultivated, and toxin induction was performed, as shown in **Figure 2B**. Total nucleic acids were extracted using phenol, as described in the section “Materials and Methods.” Ethidium bromide staining was used to visualize total nucleic acids. **(B)** Comparison of the relative abundance of 23S and 16S rRNA bands. The intensities of 23S and 16S rRNA bands were quantified using ImageJ software. The ratio of 23S rRNA/(16S+23S rRNA) and 16S rRNA/(16S+23S rRNA) was calculated and normalized to the band density of 16S+23S rRNA in the first lane (0 h). The bar graph shows a change of the values from 0 h to x h (0, 2, 4, and 6 h). **(C)** Relative densities of 5S rRNA and small RNA bands. The intensities of 5S rRNA and small RNA bands were quantified using ImageJ software, and the values were normalized to the standard in the first lane (0 h).

In many bacteria, various VapC homologs exhibit ribonuclease activity *in vitro* or *in vivo* (Ramage et al., 2009; Maezato et al., 2011; Winther and Gerdes, 2011; McKenzie et al., 2012a; Winther et al., 2013; Lopes et al., 2014). Therefore, we decided to investigate the ribonuclease activity of the Rv2019 toxin *in vivo*. First, we determined the amount of and pattern changes of total nucleic acids extracted from cells after inducing the expression of the Rv2019 toxin. The expression of the Rv2019 toxin was induced by adding L-arabinose to exponential phase cells harboring pBAD33 or pBAD33-Rv2019, and OD_600_ was measured at 2-h intervals. The same number of cells was harvested at each time point, and total nucleic acids were extracted using phenol. In the presence of L-arabinose, control cells exhibited normal exponential growth, and the level of total nucleic acids remained constant after induction (**Figure 4A**). However, after the expression of the Rv2019 toxin, we found that 23S rRNA level gradually decreased, and that more interestingly, the amount of small RNAs increased for 6 h. The intensities of the 23S and 16S rRNA and small RNA bands were analyzed using ImageJ software. **Figure 4B** shows that after the induction of the Rv2019 toxin, the levels of 23S and 16S rRNAs substantially declined after 6 h, and the levels of small RNAs increased (**Figure 4C**), indicating the accumulation of fragments of 23S and 16S rRNAs. The amount of genomic DNA at the top of the gel remained constant, indicating the RNA-specific activity of Rv2019. These results suggest that the Rv2019 toxin degrades rRNA and that its activity inhibits the growth of BL21(DE3).

### Ribonuclease Activity of Rv2019 Toxin *In vitro*

**FIGURE 5:**
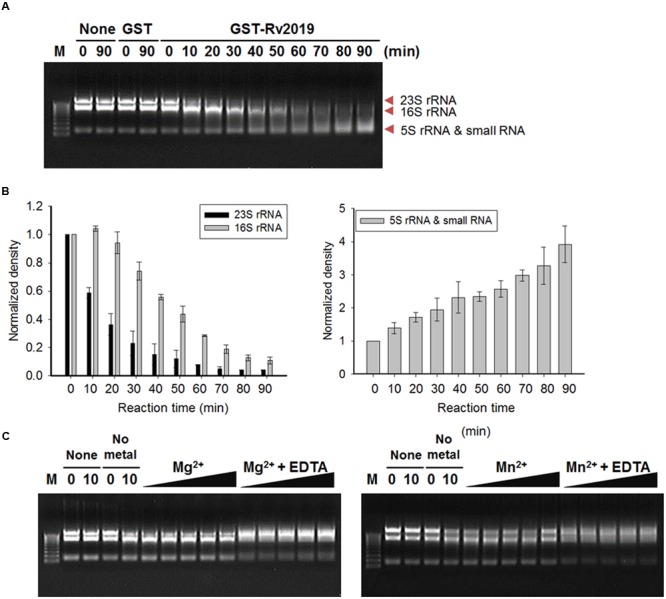
**Ribosomal RNA cleavage activity of Rv2019 *in vitro*.** Total RNAs were isolated from *E. coli* BL21(DE3) using the hot phenol method. In total, 15 pmole of GST-Rv2019 was incubated with 2 μg of total RNA in 50 mM Tris–HCl (pH 8.0). **(A)** Time-dependent ribonuclease activity of Rv2019. GST-Rv2019 was incubated with the substrate for 0 to 90 min. Cleavage assay was independently performed three times. **(B)** Relative density of RNA bands. ImageJ software was used for quantification and normalization. **(C)** Inhibition of ribonuclease activity of Rv2019 by EDTA. GST-Rv2019 was incubated for 15 min with increasing amounts of metal ion (0.0015, 0.015, 0.15, 1.5, and 15 mM) before the addition of total RNA with or without 60 mM EDTA. The reaction mixture was incubated for 10 min.

To detect the ribonuclease activity of the Rv2019 toxin *in vitro*, we cloned pGST-Rv2019, which produces a GST-Rv2019 chimera, and tested its effects on the growth of BL21(DE3) (Supplementary Figure S3B). The coexpression of GST-Rv2019 and Rv2018 recovered the normal growth of BL21(DE3) (Supplementary Figure S3C). Next, we extracted total RNAs from exponentially growing BL21(DE3) and purified GST and GST-fused Rv2019 (GST-Rv2019). Purified GST or GST-Rv2019 (15 pmoles each) was incubated with 2 μg of total *E. coli* RNAs for 90 min at 37°C in 50 mM Tris–HCl (pH 8.0). As shown in **Figure 5A**, total *E. coli* RNAs were cleaved by purified GST-Rv2019, and the activity increased with time. Moreover, RNA was not detectably digested in the absence of GST-Rv2019 or by GST. Notably, the intensity of 23S rRNA abruptly decreased after 10 min; however, 16S rRNA began to disappear after 30 min (**Figure 5B**, left) with a concomitant increase in the intensities of smaller RNAs (**Figure 5B**, right). Therefore, the purified Rv2019 toxin degrades rRNA *in vitro* as well as *in vivo*. Interestingly, the *in vitro* results showed that the Rv2019 toxin preferentially cleaved 23S rRNA. Unfortunately, although the Rv2018 antitoxin antagonized the activity of the GST-Rv2019 toxin *in vivo* (Supplementary Figure S3C), the addition of a purified N-terminal His_6_-tagged Rv2018 (antitoxin) to the reaction mixture did not inhibit the ribonuclease activity of the toxin (Supplementary Figure S4A). It is therefore very likely that improper association conditions inhibit the binding of the antitoxin to the toxin, as we confirmed the counteracting activity of His-Rv2018 to GST-Rv2019 *in vivo* (Supplementary Figure S4B).

The activities of VapC toxins of various bacteria depend on Mg^2+^, Mn^2+^, or both (Ahidjo et al., 2011; McKenzie et al., 2012a,b; Lopes et al., 2014). Therefore, we tested the effects of Mg^2+^ and Mn^2+^ on the ribonuclease activity of the Rv2019 toxin. In this assay, the reaction was incubated for 10 min instead of 90 min to detect the enhanced activity in the presence of 0.0015, 0.015, 0.15, 1.5, or 15 mM metal ions. Increasing the concentrations of metal ions did not enhance the catalytic activity, and the 23S and 16S RNAs were not digested in the presence of EDTA. When we performed the reactions in the presence of Cu^2+^, Ca^2+^, or Co^2+^, the results were the same (Supplementary Figure S4C). This is likely due to the incorporation of metal ions into the proteins during cultivation and purification, suggesting that metal ions are required for the ribonuclease activity of Rv2019 (**Figure 5C**).

### Effects of Rv2019 on mRNAs

**FIGURE 6:**
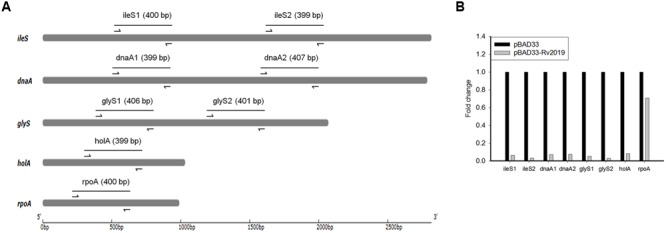
**Quantitative real-time PCR analysis of mRNAs with or without induction of Rv2019. (A)** Diagram of five genes of *E. coli* BL21(DE3) and amplicons. The arrows indicate the primers used for qRT-PCR analysis, and the lines correspond to amplicons. **(B)** qRT-PCR analysis of *ileS, dnaA, glyS, holA*, and *rpoA* mRNAs. For qRT-PCR, total RNA was extracted from BL21(DE3) harboring pBAD33 (black bars) or pBAD33-Rv2019 (gray bars) after induction for 8 h. qRT-PCR was independently performed three times, as described in the section “Materials and Methods,” and a representative result is shown. Data are shown in fold change compared to control (pBAD33).

Next, we determined whether Rv2019 digested mRNAs. For this purpose, we selected the *E. coli* BL21(DE3) genes (*ileS* and *dnaA*, ∼3 kb; *glyS*, ∼2 kb; and *holA* and *rpoA*, ∼1 kb) and used qRT-PCR to amplify specific regions of each. The expression of the Rv2019 toxin was induced by adding L-arabinose to exponential phase cells harboring pBAD33 or pBAD33-Rv2019, and total RNAs were extracted after induction for 8 h. To amplify specific regions of five mRNAs, we designed primer sets that generate an approximately ∼400-bp amplicon (**Figure 6A**). The cDNA synthesized from total RNA was subjected to qRT-PCR, and the differences in relative expression for each gene are shown in **Figure 6B**. The relative mRNA amounts of ileS1, ileS2, dnaA1, dnaA2, glyS1, glyS2, and holA were significantly lower (∼9-fold) when the Rv2019 toxin was expressed. However, the transcript amount of rpoA was marginally lower in the presence of the Rv2019 toxin, suggesting that Rv2019 may not cleave the *rpoA* transcript. Therefore, our results show that *ileS, dnaA, glyS*, and *holA* mRNAs serve as substrates for the Rv2019 toxin and that the seven specific regions of these mRNAs contain the sequence cleaved by Rv2019. Based on these results, mRNA was likely digested by the ribonuclease activity of the Rv2019 toxin.

### Assessment of the Toxic Effects of 5 *Mtb* Toxins on *M. smegmatis*

**FIGURE 7:**
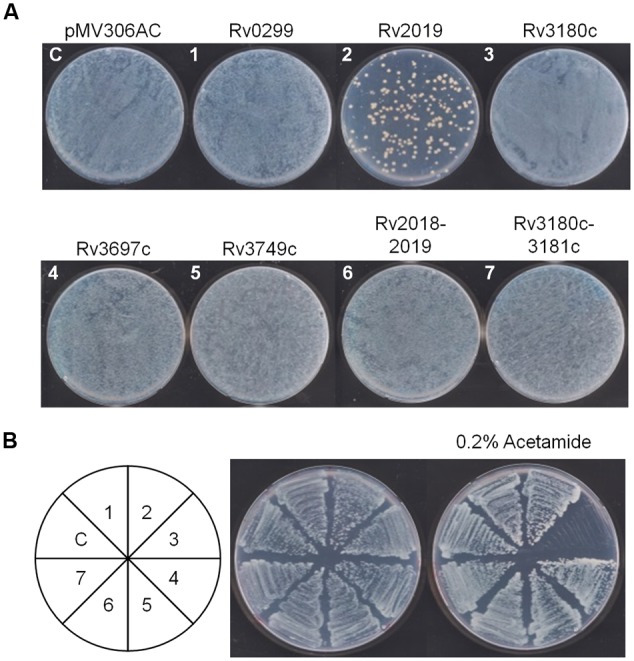
**Toxic effect of the five toxins on the growth of *M. smegmatis*. (A)** Transformation of pMV306AC-toxin plasmid into *M. smegmatis* mc^2^ 155. One microgram of DNA was electrophorated and cells were plated on Middlebrook 7H10 solid media. The plates were incubated for 3 days at 37°C. **(B)** Effect of toxin on the induction on colony formation of *M. smegmatis* mc^2^ 155. Each transformant from **(A)** was grown for 3 days at 37°C. The culture was diluted 100-fold into Middlebrook 7H9 liquid media and streaked on Middlebrook 7H10 solid media in the absence and presence of 0.2% acetamide. The numbers in the circle correspond to those in **(A)**.

So far, we determined the toxicities of five putative *Mtb* toxins to *E. coli* and found that only the Rv2019 toxin caused toxicity, presumably through its ribonuclease activity. Therefore, we assessed the toxic effects of five candidate *Mtb* TA systems on a non-pathogenic species *M. smegmatis*, which is closely related to *Mtb*. For this purpose, we constructed expression vectors in pMV306AC, which harbors an acetamide-inducible promoter and integrates into the *attB* site of *M. smegmatis* genomic DNA. The resulting plasmids pMV306AC-Rv2019, pMV306AC-Rv2018-2019, pMV306AC-Rv3697c, pMV306AC-Rv3180c, pMV306AC-Rv3180c-3181c, pMV306AC-Rv0299, and pMV306AC-Rv3749c were individually transformed and integrated into the genome of *M. smegmatis*, as described in the section “Materials and Methods.” The initial transformation results showed that the transformation efficiency of pMV306AC-Rv2019 was significantly lower than those of the other vectors (**Figure 7A**, plate 2). However, the transformation of pMV306AC-Rv2018-2019 resulted in the similar colony number with that obtained from control vector (**Figure 7A**, plate 6). This suggests that transient expression of Rv2019 is toxic to *M. smegmatis* and this toxicity was reverted by the expression of antitoxin, Rv2018. Interestingly, when we streaked colonies formed on the initial plates, only the Rv3180c toxin inhibited colony formation of *M*. *smegmatis* in the presence of 0.2% acetamide (**Figure 7B**, sector 3). This toxicity was neutralized by the antitoxin Rv3181c (**Figure 7B**, sector 7). Further, we found that only the Rv3180c toxin inhibited the growth of *M*. *smegmatis* after the addition of acetamide to cells cultured in liquid Middlebrook 7H9 containing 0.2% glycerol and 0.02% Tween 80 (data not shown). Peculiarly, however, the cells transformed with pMV306AC-Rv2019 did not exhibit an inhibitory phenotype upon induction (**Figure 7B**, sector 2). These findings raise the possibility that mutation, rearrangement, or both of the promoter or the locus harboring *Rv2019* prevents the expression of Rv2019, and sequencing analysis revealed that transformants of pMV306AC-Rv2019 harbor several spontaneous mutations in the open reading frame of *Rv2019* (data not shown). Therefore, our results demonstrate that the expression of Rv2019 and Rv3180c inhibited the growth of *M*. *smegmatis*, and their cognate antitoxins counteracted the toxicities of toxins.

## Discussion

*Mycobacterium tuberculosis* possesses 50 putative VapBC TA systems. The structures of the complexes formed by VapBC3, VapBC5, VapBC15, and VapBC30 have been determined (Miallau et al., 2009; Min et al., 2012; Das et al., 2014; Lee et al., 2015). The active sites of VapC toxins consist of three or four highly conserved acidic residues that bind to Mg^2+^, Mn^2+^, or both, which is a characteristic of the PIN domain. These divalent metal ions coordinate with acidic residues and form a concave active site, thereby helping to stabilize the interaction with negatively charged RNA that is required for RNase activity (Ahidjo et al., 2011; McKenzie et al., 2012a,b; Lopes et al., 2014).

When expressed in *E. coli*, the VapC homolog Rv2019 toxin, which triggered a growth defect, was neutralized by its cognate antitoxin Rv2018. The Rv2019 toxin appeared to possess ribonuclease activity using free rRNA as a substrate *in vivo* and *in vitro*. The Rv2019 toxin cleaved rRNA less effectively *in vivo* than *in vitro*, likely due to the association of the rRNA with ribosomal proteins *in vivo*. Interestingly, the digestion rate of 23S rRNA was much faster than that of 16S rRNA, indicating more preferred target sites on 23S rRNA. Presumably, this RNase activity is either sequence- or conformation-specific, nevertheless, it is likely that cleavage site specificity of Rv2019 is quite low, resulting in small RNA accumulation. However, this activity was inhibited in the presence of EDTA. Metal ion dependence was further supported by analysis of the mutants Rv2019_E41K_ and Rv2019_D98K_ (**Figure 2C**). Interestingly, in *M. smegmatis*, initial transformation of pMV306AC-Rv2019 negatively affected the colony formation, yielding the substantially low transformation efficiency of pMV306AC-Rv2019, meanwhile, this effect was masked by the expression of Rv2018 (**Figure 7A**). This demonstrates that Rv2019 is paired with Rv2018. Therefore, our findings demonstrate that Rv2019 is a *bona fide* VapC toxin.

Among five putative toxins, only RV2019 inhibited colony formation after its expression was induced in *E. coli*, which prompted us to determine the induction level of each recombinant protein. Unfortunately, we were unable to detect significant protein induction using SDS-PAGE (data not shown), nevertheless, the low expression level of Rv2019 was sufficient to cause toxicity.

We next attempted to express GST-fused toxin proteins to determine whether a higher level of expression induced a different phenotype of cell growth. The fusion of each toxin with GST-tag apparently increased the level of induction (Supplementary Figure S3A), and we detected the same toxic phenotype of cells that expressed GST-Rv2019. Notably, serial dilutions of cells that expressed GST-Rv0299 formed less dense colonies, suggesting that GST-Rv0299 was marginally toxic (Supplementary Figure S3B). In *E. coli* MazF, there are seven amino acid residues, Asp16, Asp18, Glu24, Arg29, Gly66, Asp76, and Arg86 that are involved in the substrate binding, and out of these seven residues, Glu24 and Arg29 are pivotal for catalytic activity of MazF. However, Rv2099 conserved only Asp16 and Glu24 (residue numbers as in *E. coli* MazF), which might explain the marginal toxicity (Li et al., 2006). Therefore, further studies are required to determine the mechanism of toxicity of Rv2099 as a putative MazF homolog.

Interestingly, compared with the results shown in **Figure 2**; Supplementary Figure S3B, the expression of C-terminal His_6_-Rv2019 toxin exhibited a reduced toxic effect (Supplementary Figure S2B). This is probably why the active site is sterically hindered by the His-tag at the C-terminal region which points the incurvate catalytic site (Miallau et al., 2009; Min et al., 2012; Das et al., 2014; Lee et al., 2015).

Type II TA families were originally classified according to their amino acid sequences and three-dimensional structural similarities of their respective toxins (Pandey and Gerdes, 2005). Further, each toxin was paired with its cognate antitoxin depending on their specific interactions, e.g., VapBC and MazEF (Zhang J. et al., 2003; Lopes et al., 2014). Among VapC homologs containing the PIN domain, the majority of toxins interact with antitoxins containing a ribbon–helix–helix (RHH) domain. However, several antitoxins with PHD, AbrB, COG2442, COG2886, MerR, or Xre domains interact with the VapC toxin (Makarova et al., 2009).

In the Rv3180c-Rv3181c TA system, the Rv3180c toxin, which contains a PIN domain, has been predicted to possess ribonuclease activity, and the Rv3181c antitoxin possesses a PHD domain that is recently expected to associate with PIN domain toxins (Makarova et al., 2009). Therefore, the antitoxin containing a PHD domain was considered to interact with a toxin containing the PIN domain. Moreover, it has recently been revealed that a toxin and non-cognate antitoxin interact, and therefore, TA families are independently divided into 12 super-families of Type II toxins and 20 super-families of Type II antitoxins, respectively (Leplae et al., 2011).

There are potentially nine TA systems (one MazF homolog, one VapC homolog, and seven putative TA systems) in *M. smegmatis* and 33 TA modules in *E. coli* (Shao et al., 2011; Yamaguchi et al., 2011). It should be noted that neither native nor highly expressed GST-fused Rv3180c was toxic to *E. coli* (Supplementary Figure S3), suggesting a lack of relationship between expression level and toxicity. Unlike in *E. coli, M. smegmatis* cells could not form colonies upon induction of Rv3180c.

Therefore, it is possible that Rv3180c may be neutralized by non-cognate antitoxins present in *E. coli* or that structural conformation of Rv3180c is different in *E. coli* and *M. smegmatis*.

In the present study, we cloned 5 putative *Mtb* TA modules and expressed them in *E. coli* and *M. smegmatis*. Our results revealed that the novel toxin Rv2019 is an RNase that degrades rRNAs and mRNAs and that the Rv2019 and Rv3180c toxins and their cognate Rv2018 and Rv3181c antitoxins functionally associate, respectively. Further studies are required to characterize the other four TA systems. Considering the vast numbers of TA systems of the *M. tuberculosis* complex, including *Mtb*, such studies promise to contribute to a better understanding of the importance of *Mtb* TA systems for controlling latency, persistence, biofilm formation, and stress response.

## Author Contributions

YK: executing experiments. EC: writing manuscript. JH: Guiding YK and EC.

## Conflict of Interest Statement

The authors declare that the research was conducted in the absence of any commercial or financial relationships that could be construed as a potential conflict of interest.
