# Supplementary material for: Functional Studies of Five Toxin-Antitoxin Modules in Mycobacterium tuberculosis H37Rv
Source: Front Microbiol. 2016 Dec 21;7:2071. doi: 10.3389/fmicb.2016.02071 (PMC5175181; doi:10.3389/fmicb.2016.02071)
Supplement: Supplementary file 1 [file Presentation_1.PDF]

***Supplementary Material***

**The Rv2019 toxin of *Mycobacterium tuberculosis* H37Rv as a VapC-like ribonuclease**

Yoonji Kim[†], Eunsil Choi[†], and Jihwan Hwang*

*** Correspondence:** Dr. Jihwan Hwang: hwangjh@pusan.ac.kr

## Fig. S1

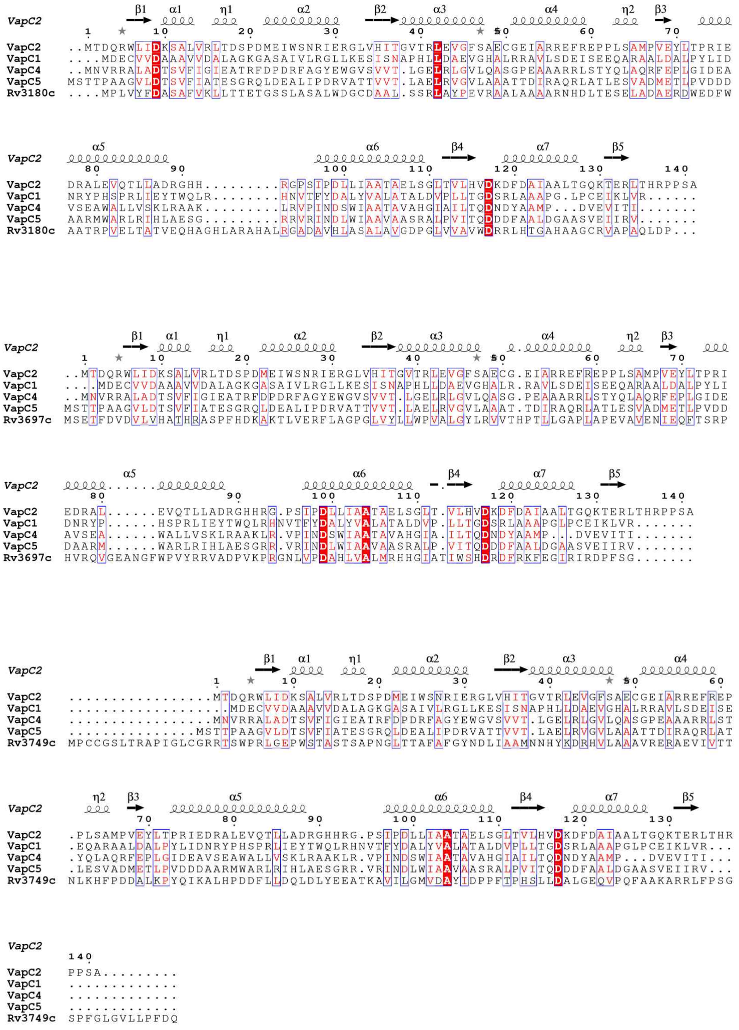

**Fig. S1** Sequence alignments of *Mtb* VapC homologs with Rv3180c, Rv3697c or Rv3749c. Alignment analysis was carried out with the ESPript program. Identical residues are shown in black boxes and similar residues in white boxes. The numbers correspond to the residue numbers. VapC1, *Mtb* VapC1 (GenBank accession no. CCP42788.1); VapC2, *Mtb* VapC2 (CCP43031.1); VapC4, *Mtb* VapC4 (CCP43334.1); VapC5, *Mtb* VapC5 (CCP43368.1); Rv3180c, *Mtb* Rv3180c (NP_217696.1); Rv3697c, *Mtb* Rv3697c (NP_218214.1); Rv3749c, *Mtb* Rv3749c (NP_218266.1).

## Fig. S2

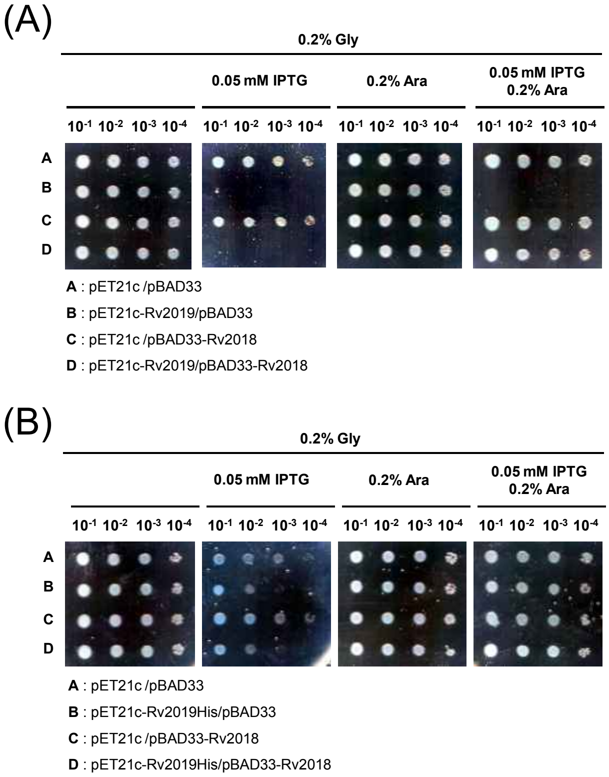


**Fig. S2** Neutralization of toxic effect of Rv2019 toxin by Rv2018 antitoxin. **(A)** BL21(DE3) harboring two compatible plasmids (pET21c & pBAD33, pET21c-Rv2019 & pBAD33, pET21c & pBAD33-Rv2018 or pET21c-Rv2019 & pBAD33-Rv2018) was diluted at exponential phase. Then the cells were harvested by centrifugation, resuspended to an $OD_{600}$ of 0.2 in M9 minimal media and serially diluted ($10^{-1}$, $10^{-2}$, $10^{-3}$ and $10^{-4}$). Each diluted sample was spotted on M9 agar plates. **(B)** BL21(DE3) harboring two compatible plasmids (pET21c & pBAD33, pET21c-Rv2019His & pBAD33, pET21c & pBAD33-Rv2018 or pET21c-Rv2019His & pBAD33-Rv2018) was diluted at exponential phase. Then the cells were spotted on M9 agar plates as in (A).

**Fig. S3**

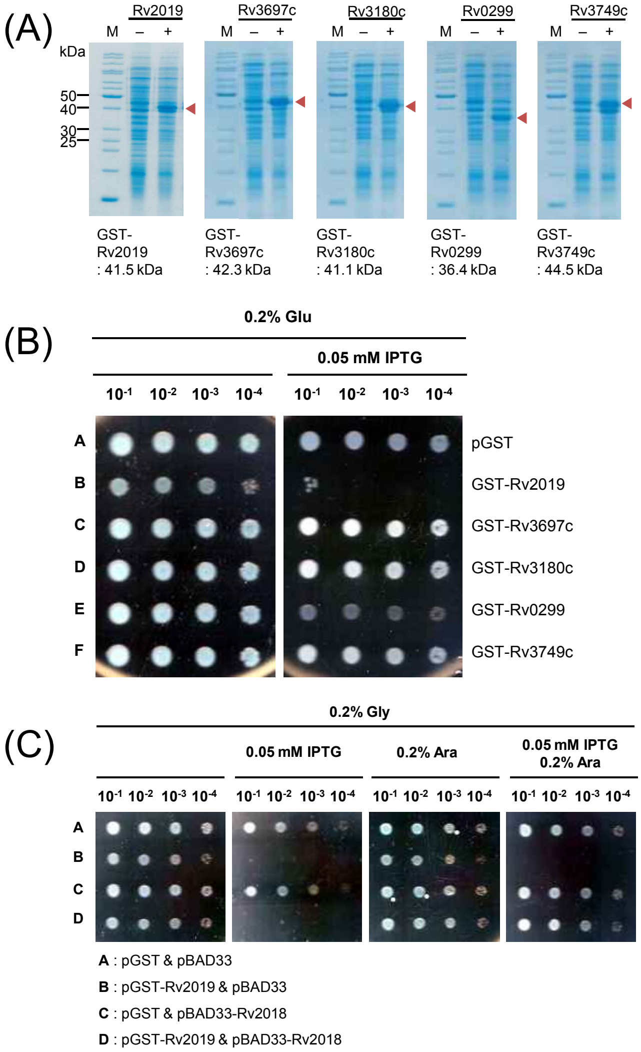


**Fig. S3 (A)** Expression of GST-fused toxin. BL21(DE3) harboring pGST-toxin (Rv2019, Rv3697c, Rv3180c, Rv0299, or Rv3749c) was grown at exponential phase. Then IPTG was added at a final concentration of 0.05 mM and the cultures were further incubated with shaking (200 rpm) at 37°C for 1 h. GST-fused toxin proteins were separated on 15% polyacrylamide gel and stained with Coomassie Blue. M, protein molecular weight standard; -, uninduced culture; +, induced culture. **(B)** The effect of GST-toxin on colony formation. BL21(DE3) harboring pGST (control) or pGST-toxin (Rv2019, Rv3697c, Rv3180c, Rv0299 or Rv3749c) was diluted at exponential phase. Then the cells were harvested by centrifugation, resuspended to an $OD_{600}$ of 0.2 in M9 minimal media and serially diluted ($10^{-1}$, $10^{-2}$, $10^{-3}$ and $10^{-4}$). Each diluted sample was spotted on M9 agar plates. **(C)** Neutralization of toxic effect of Rv2019 toxin by Rv2018 antitoxin. BL21(DE3) harboring two compatible plasmids (pGST & pBAD33, pGST-Rv2019 & pBAD33, pGST & pBAD33-Rv2018 or pGST-Rv2019 & pBAD33-Rv2018) was diluted at exponential phase. Then the cells were spotted on M9 agar plates as in (B).

**Fig. S4**

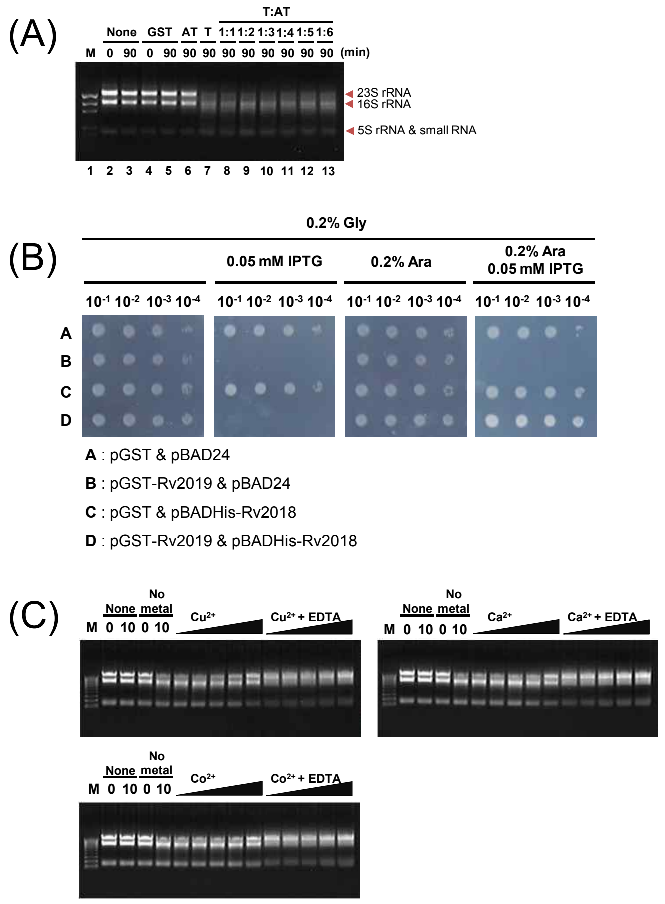


**Fig. S4 (A)** Ribosomal RNA cleavage activity of Rv2019 in the presence of Rv2018. Total RNAs were isolated from *E. coli* BL21(DE3) using the hot phenol method. 15 pmole of GST-Rv2019 (lane 7) or 90 pmole of $His_6$-Rv2018 (lane 6) was incubated with 2 μg of total RNA in 50 mM Tris–HCl (pH 8.0). 15 pmole of GST-Rv2019 was incubated for 15 min with increasing amounts of $His_6$-Rv2018 (15, 30, 45, 60, 75, and 90 pmole) before the addition of total RNA (lanes 8-13). The reaction mixture was incubated for 90 min. **(B)** Neutralization of the toxic effect of GST-Rv2019 by the His-Rv2018 antitoxin. The experiment was carried out as in Fig. 3. **(C)** Inhibition of ribonuclease activity of Rv2019 by EDTA. GST-Rv2019 was pre-incubated for 15 min with increasing amounts of metal ion (0.0015, 0.015, 0.15, 1.5, and 15 mM) before the addition of total RNA with or without 60 mM EDTA. The reaction proceeded for 10 min.

## Table. S1

| Plasmid | Primer | Sequences |
|---|---|---|
| pBAD33-Rv2019 | Rv2019_F | 5'-AACATATGCAGCCTGATCGGAATCTC-3' |
| | Rv2019_R | 5'-AAAAGCTTCTAGAGCACGCGAACGA-3' |
| pBAD33-Rv3697c | Rv3697c_F | 5'-AACATATGAGCGAAACCTTTGACGTCGA-3' |
| | Rv3697c_R | 5'-AAAAGCTTTCAGCCGGAGAAGGGGTC-3' |
| pBAD33-Rv3180c | Rv3180c_F | 5'-AACATATGCCGCTCGTCTACTTCGACG-3' |
| | Rv3180c_R | 5'-AAAAGCTTTCACGGGTCGAGTTGGGC-3' |
| pBAD33-Rv0299 | Rv0299_F | 5'-AGCTCACCCTTCGTCTGGCT-3' |
| | Rv0299_R | 5'-AAAAGCTTCTAACAGAGCAGAGCTGTCACG-3' |
| pBAD33-Rv3749c | Rv3749c_F | 5'-AACATATGCCCTGTTGCGGTTCG-3' |
| | Rv3749c_R | 5'-AATCTAGACTATTGATCGAATGGGAGCA-3' |
| pET21c-Rv2018 | Rv2018_F | 5'-CACTATGCGCGTCACGATGG-3' |
| | Rv2018_R | 5'-GCCTGGATGGTGTCAACGGT-3' |
| pET21c-Rv3697A | Rv3697A_F | 5'-ACGACCTGCTGTTGATCGTC-3' |
| | Rv3697A_R | 5'-CCAGACGCAGTTGCTGAAGA-3' |
| pET21c-Rv3181c | Rv3181c_F | 5'-ATTCTTACCTTGAATAAATCTGGCATTGG-3' |
| | Rv3181c_R | 5'-CGAAAGCTTCCAGTTGTTCAGC-3' |
| pET21c-Rv0298 | Rv0298_F | 5'-AACATATGACGAAAGAGAAGATCTCCGTGACG-3' |
| | Rv0298_R | 5'-AAGAATTCTCAACTTCCGGCCGCCCG-3' |
| pET21c-Rv3750c | Rv3750c_F | 5'-AACATATGACCTCCTTGCTGGAGGTG-3' |
| | Rv3750c_R | 5'-AAGAATTCCTACTTCTTTTCTGCAAGCG-3' |
| | D4K_F | 5'-AACATATGCAGCCTAAGCGGAATCTC-3' |
| | E41K_F | 5'-GACAACGATGCGGAAGCACTACGGCGAGAC-3' |
| | E41K_R | 5'-GTCTCGCCGTAGTGCTTCCGCATCGTTGTC-3' |
| | D98K_F | 5'-CTATTCTGTGTGCCGCGGGCCAAGATC-3' |
| | D98K_R | 5'-GATCTTGGCCCGCGGCACACAGAATAG-3' |
| **Gene** | **Primer for qRT-PCR** | **Sequences** |
| *ileS* | ileS1 (Forward) | 5'-CGTTGACTGCCGTTCTGCGC-3' |
| | ileS1 (Reverse) | 5'-ATCGCCGAGGATTGCCGGAA-3' |
| | ileS2 (Forward) | 5'-ACCAGCACCGTGGTTGGTTC-3' |
| | ileS2 (Reverse) | 5'-CCTGTGCCGCTTTCGCACAA-3' |
| *dnaA* | dnaA1 (Forward) | 5'-CCTCTGATAACATTCCTGGCGTA-3' |
| | dnaA1 (Reverse) | 5'-CTGGTGCTTCGTCTGCAACA-3' |
| | dnaA2 (Forward) | 5'-AGCTCACCCTTCGTCTGGCT-3' |
| | dnaA2 (Reverse) | 5'-AAACGCCTGGCGGATACGAC-3' |
| *glyS* | glyS1 (Forward) | 5'-CTCTGGCGAAACTGCCGATC-3' |
| | glyS1 (Reverse) | 5'-GGCACCGCGAGGAATTTCTC-3' |
| | glyS2 (Forward) | 5'-CACTATGCGCGTCACGATGG-3' |
| | glyS2 (Reverse) | 5'-GCCTGGATGGTGTCAACGGT-3' |
| *holA* | holA (Forward) | 5'-ACGACCTGCTGTTGATCGTC-3' |
| | holA (Reverse) | 5'-CCAGACGCAGTTGCTGAAGA-3' |
| *rpoA* | rpoA (Forward) | 5'-ATTCTTACCTTGAATAAATCTGGCATTGG-3' |
| | rpoA (Reverse) | 5'-CGAAAGCTTCCAGTTGTTCAGC-3' |
